# Emergence of Chronic Lymphocytic Leukemia During Admission for COVID-19: Cause or Coincidence?

**DOI:** 10.7759/cureus.23470

**Published:** 2022-03-24

**Authors:** Prachi Saluja, Nitesh Gautam, FNU Amisha, Mazin Safar, Thaddeus Bartter

**Affiliations:** 1 Department of Internal Medicine, University of Arkansas for Medical Sciences, Little Rock, USA; 2 Department of Hematology and Medical Oncology, University of Arkansas for Medical Sciences, Little Rock, USA; 3 Department of Pulmonary and Critical Care Medicine, University of Arkansas for Medical Sciences, Little Rock, USA

**Keywords:** chronic lymphocytic leukemia (cll), severe acute respiratory syndrome coronavirus -2 (sars-cov-2), hemato-oncology, pneumonia, covid-19 pneumonia, covid-19

## Abstract

Chronic lymphocytic leukemia (CLL) is the most common leukemia affecting the western adult population. While CLL is known to be a risk factor for morbidity and mortality from coronavirus disease 2019 (COVID-19), COVID-19 has not been shown to be a risk factor for the development of CLL. We report a case of a 55-year-old man who presented with COVID-19 pneumonia and developed overt CLL during hospitalization. Four other cases were culled from the literature. We discuss mechanistic possibilities for the unmasking of CLL in susceptible individuals with COVID-19.

## Introduction

Patients with hematological malignancies are particularly at risk when infected with the severe acute respiratory syndrome coronavirus-2 (SARS-CoV-2), with case fatality rates approaching 34% due to inadequate immune responses [1]. Chronic lymphocytic leukemia (CLL) is the most common leukemia affecting the western adult population [2], and there are several reports elaborating on the disease course in CLL patients. While CLL is known to be a risk factor for morbidity and mortality from coronavirus disease 2019 (COVID-19), COVID-19 has not been shown to be a risk factor for the development of CLL. We report a case of a 55-year-old man who presented with COVID-19 pneumonia and developed overt CLL during hospitalization. Four other cases were culled from the literature. The data raise the question of whether COVID-19 infection can unmask CLL in susceptible individuals.

## Case presentation

**Figure 1:**
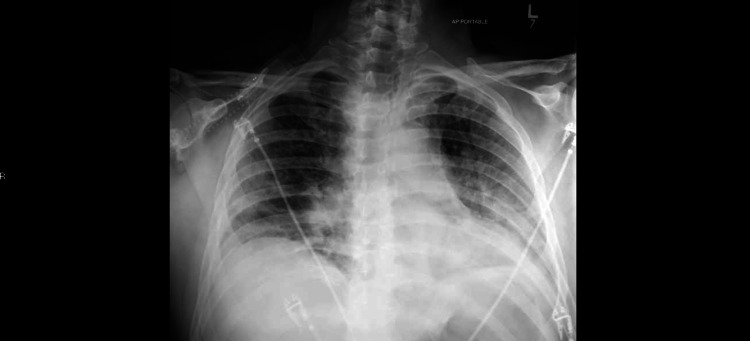
Chest X-Ray on the day of admission

A 55-year-old man with a past medical history of hypertension and hypothyroidism presented to the hospital after 8 days of shortness of breath, dry cough, and fever. His COVID-19 PCR test came back positive. He was tachypneic and hypoxic on arrival, requiring oxygen support via a venturi mask. His temperature was 100.1°F, blood pressure 124/79 mmHg, and pulse 74 beats per minute. Initial laboratory workup included neutrophilic leukocytosis of 12k with normal lymphocyte, hemoglobin, and platelet counts. The metabolic panel revealed hyponatremia with a serum sodium of 131 mmol/L, and mild hypochloremia. Inflammatory markers, including d-dimer, fibrinogen, erythrocyte sedimentation rate (ESR), C-reactive protein (CRP), and ferritin were all elevated, while pro-calcitonin was within normal limits. Chest X-ray demonstrated bilateral airspace disease (Figure 1).

**Figure 2:**
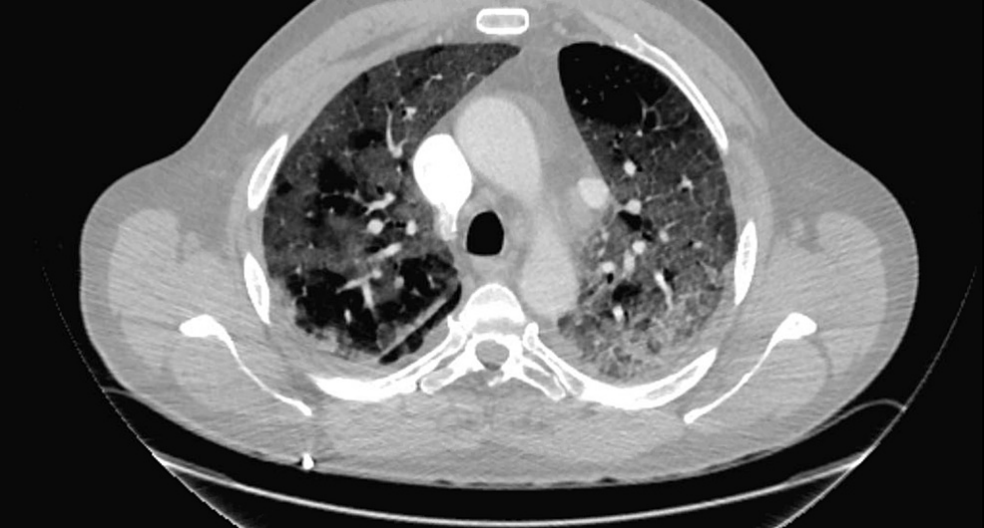
CT scan of the chest on day 7 of admission

COVID-19 was treated initially with dexamethasone and remdesivir, with the addition of antibiotics for possible bacterial pneumonia. The patient received a dose of tocilizumab on day 3 of hospitalization. He suffered a progressive decline, with worsening oxygenation. A computed tomographic (CT) scan of the chest on day 7 of admission showed acute pulmonary emboli and extensive bilateral parenchymal disease (Figure 2). He was anticoagulated, and steroid dosing was increased due to concern for post-infectious organizing pneumonia. His illness progressed and included bilateral pneumothoraxes and noninvasive ventilation followed by intubation. He succumbed to his illness on day 29 of hospitalization.

**Figure 3:**
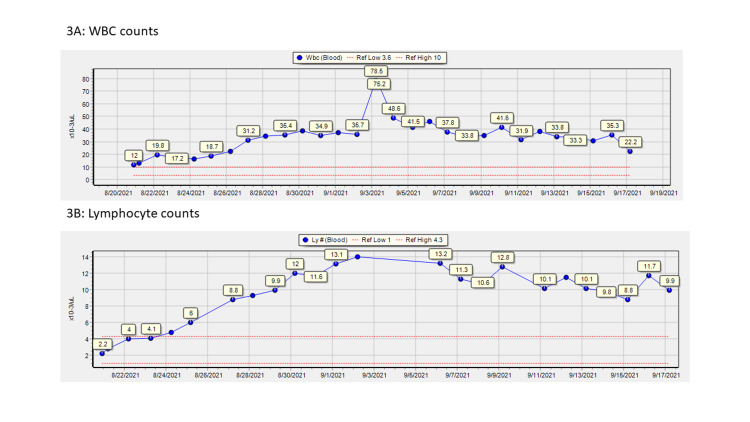
Leukocyte counts during admission WBC: white blood cells

Over the course of his stay, he had developed progressive lymphocytosis (peaked at 14 × 10^3^/µL) and neutrophilia (peaked at 32 × 10^3^/µL), with white blood cell (WBC) count peaking at 78.5 × 10^3^/µL on day 15 of admission (Figure 3). A peripheral smear done early during hospitalization (day 9) had shown leukocytosis with neutrophilia and lymphocytosis without any myelocytes or blasts. A repeat smear on day 15 of admission showed smudge cells concerning for CLL. Flow cytometry of peripheral blood obtained on day 16 was diagnostic for CLL; it showed malignant cells expressing CD45, CD19, CD5, CD20, CD22, CD23, and kappa light chain. Malignant cells were negative for expression of CD10, FMC7, CD79B, and CD38.

## Discussion

**Figure 4:**
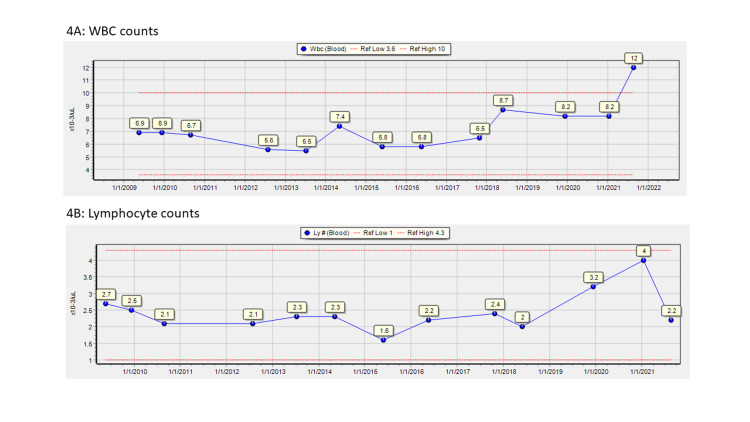
Leukocyte data 2010–admission WBC: white blood cells

The data are consistent with the concept that COVID-19 infection caused or unmasked CLL. Ten years of pre-admission data, shown in Figure 4, had shown no evidence of lymphocytosis, and admission data showed no evidence of CLL. Subsequent data during this patient’s hospital stay differed from those on admission and were diagnostic of CLL.

The concept of infection as a precipitant for CLL is not new. In two large population studies, Landgren et al. demonstrated that prior cases of pneumonia and chronic inflammatory conditions were significant risk factors for subsequent CLL. The two most significant risk factors for CLL in their populations were pneumonia in the preceding year (OR: 5.9, 95% CI: 4.5-7.7) and multiple cases of pneumonia (p<0.001 for the trend) [3,4]. Similarly, there have been studies reporting increased use of antimicrobials in the preceding 6-10 years among future CLL patients [5].

**Figure 5:**
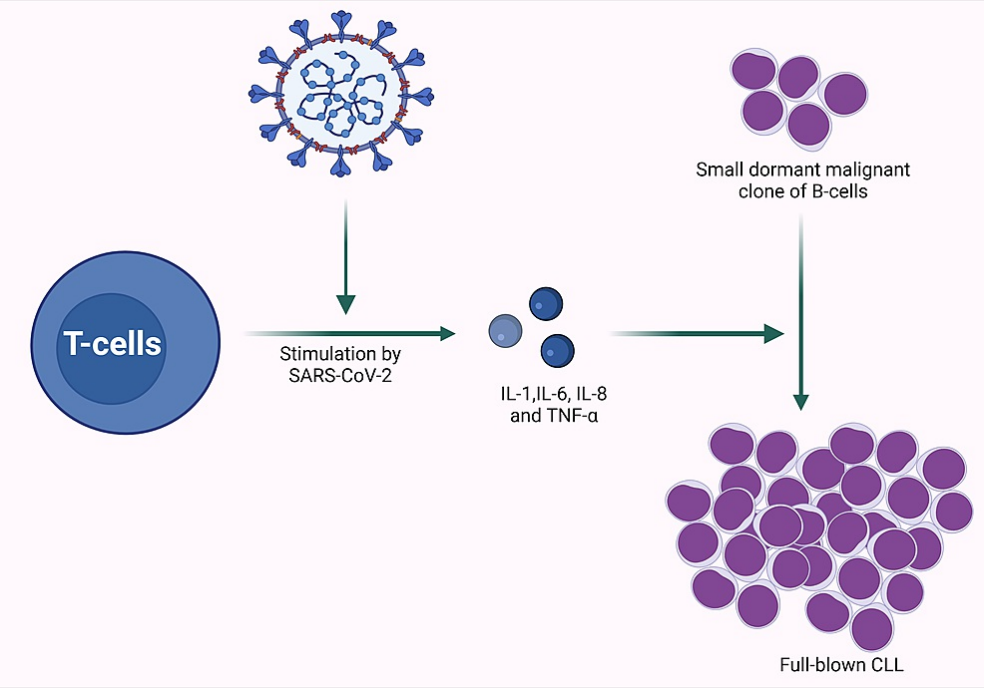
Graphic representation of SARS-CoV-2 stimulating cytokine production, possibly leading to unmasking of CLL CLL: chronic lymphocytic leukemia; TNF: tumor necrosis factor; SARS-CoV-2: severe acute respiratory syndrome coronavirus-2 Created with BioRender.com

With the advent of immunotherapy, it has become evident that many malignancies are associated with and even infiltrated by immune cells that have the capacity to recognize and attack the malignant population but that have been “silenced;” immunotherapy potentiates this innate and already-present capacity [6]. If a silenced immune response can be activated, it is likely that the opposite can occur, that an effective immune response can be inactivated, allowing “escape” of a malignancy whose genetic substrate had been dormant. This mechanistic understanding is supported by the cited literature and by this case. Given the rapidity with which CLL emerged in this case, it is far more likely that the acute inflammatory response to COVID-19 infection broached a previously effective immune control than that the patient developed and expressed the required mutation over the course of two weeks (Figure 5).

A Medline search was performed with the goal of finding other case reports of CLL newly diagnosed during acute COVID-19 infection. The keywords “Leukemia, Lymphocytic, Chronic, B-Cell”[Mesh] OR “Leukemia, Prolymphocytic, T-Cell”[Mesh] OR “CLL” AND “COVID-19” led to the identification of four case reports [7-10]. The available data, this case included, are presented in Table 1. Documentation was not thorough enough to rule out the possibility that these additional cases may simply reflect rare instances of the serendipitous diagnosis of CLL during COVID-19 infection; there are a few reported cases in which patients with established CLL developed “Covid-induced lymphocytosis” [11]. Some of the data, however, are consistent with the mechanistic understanding proposed for this case. The median age for diagnosis of CLL is 72, and two of the cases, like the case presented herein, were unusually young at 49 and 55 years of age, consistent with (though far from diagnostic of) the concept of early unmasking due to an infectious trigger [7,9]. The case presented by Largeaud et al.’s study was of an 83-year-old gentleman, but the authors provided evidence of a normal WBC count both one year before and at the time of admission, lymphocytosis with studies diagnostic of CLL on day 11 of admission, and subsequent resolution of lymphocytosis 5 months after discharge although the CLL clone was still detectable [10]. The case is consistent with the hypothesis of immune escape during infection and some improvement in immunosurveillance after the acute infection. Further epidemiological studies are needed to determine causality, if any, between COVID-19 infection and the occurrence of CLL.

**Table 1:** Reported cases of discovery of CLL during COVID-19 admission CLL: chronic lymphocytic leukemia; COVID-19: coronavirus disease 2019

Author	Age of index patient (years)	Gender of index patient	Comorbidities	Reported day of hospitalization with maximum lymphocytes	Lymphocyte count (Ly #)	Any lymphadenopathies reported	Positivity on flow cytometry OR Immunophenotype results	Death or Follow-up
This Case	55	Male	Hypertension and hypothyroidism	Day 15	14 × 10^3^/µL	Mediastinal lymphadenopathy	Positive for CD45, CD19, CD5, CD20, CD22 (dim), CD23, and kappa light chain	In-hospital mortality 29 days after admission
Largeaud et al. (Letter to the editor) [10]	83	Male	Radiotherapy-treated lung cancer	~Day 30	~40 × 10^3^/L	Retroperitoneal and mediastinal lymphadenopathy	CD5 positive	At 5 months, resolution of lymphocytosis, but CLL-type-clone detectable
Ali et al. [7]	49	Male	None	Day 1	26.7 × 10^3^/µL	None documented on exam	Positive for CD19, CD5, CD23, CD20 (dim), CD43, CD200 with a dim expression of kappa light chain restriction and FMC7	At 1 month, plan for observation for early stage CLL
Jerez and Ernst [9]	55	Male	None	Day 1	7.8 × 10^3^/µL	No comment	Positive for CD23, CD200, with dim expression of CD20 and CD43	No comment
Charra et al. [8]	76	Male	Surgically treated colon cancer	Day 7	154 × 10^3^/µL	Multiple adenopathies at presentation	Immunophenotyping showed B-cells expressing one light chain Immunoglobulin	Death after 10 days of admission

## Conclusions

We posit that the present case report is of interest not because of its rarity but because of the possible mechanistic implications. The case underscores deficiencies in our knowledge with respect to the development or revelation of malignancy during an acute inflammatory process. COVID-19 stimulates an intense immune response, with elevated levels of pro-inflammatory cytokines such as interleukin (IL)-1, IL-6, IL-8, and tumor necrosis factor (TNF)-α. We believe that the cited literature and this case are consistent with a mechanistic understanding that a clone with malignant potential that had previously been controlled by effective immunosurveillance can be unmasked by immunologic modulations caused by infection/inflammation. In the presented case, that trigger was hypothesized to be acute COVID-19 infection.
